# Posterior Reversible Encephalopathy Syndrome Caused by Fioricet (Butalbital-Acetaminophen-Caffeine)

**DOI:** 10.1155/2019/5410872

**Published:** 2019-07-11

**Authors:** Naomi Cret, Madalina Halalau, Shahab Rezvani, Alexandra Halalau

**Affiliations:** ^1^Wayne State University, Detroit, Michigan, USA; ^2^Neuroradiology Department, Beaumont Hospital, Royal Oak, Michigan, USA; ^3^Oakland University William Beaumont School of Medicine, Rochester, Michigan, USA; ^4^General Internal Medicine Division, Beaumont Hospital, Royal Oak, Michigan, USA

## Abstract

A common side effect of Fioricet (butalbital-acetaminophen-caffeine) is high blood pressure caused by the caffeine content. We report a case of a 54-year-old female who developed the worst headache of her life after taking 2 tablets of butalbital-acetaminophen-caffeine every six hours for three days before presenting to the emergency department, where her blood pressure was 178/87 mmHg. A brain MRI showed edema in the subcortical white matter of the right occipital lobe, right parietal lobe, and left occipital lobe. Posterior reversible encephalopathy syndrome (PRES) was diagnosed. The patient returned within the week with severe headaches, visual hallucinations, and a blood pressure of 150/80 mmHg. Repeat brain MRI showed slight improvement of edema. Her brain imaging studies completely normalized at 10 months after diagnosis. Although the patient received appropriate treatment, she was unable to make full recovery. Due to her inability to work and the constant pain, the patient continues to struggle with depression.

## 1. Introduction

Posterior reversible encephalopathy syndrome (PRES) is a neurotoxic state that occurs secondary to the inability of the posterior circulation to autoregulate in response to acute changes in blood pressure [1]. It can be triggered by endothelial dysfunction caused by circulating endogenous or exogenous toxins. Hyperperfusion with resultant disruption of the blood-brain barrier or circulating toxins result in vascular leakage and vasogenic edema formation, but not infarction, most commonly seen in the parieto-occipital regions [2]. PRES is also known as acute hypertensive encephalopathy or reversible posterior leukoencephalopathy. While the incidence of PRES is unknown, it has been seen in patients ranging from 2 to 90 years of age, with the syndrome being more common in women [3].

This entity has been described mainly related to hypertension, renal failure, eclampsia, sepsis and multiorgan failure, autoimmune diseases, the use of chemotherapy, immunosuppressive (tacrolimus, cyclosporine, and chemotherapeutics), and illicit drugs (cocaine), or organ transplantation [4–6]. PRES is characterized by rapid onset of symptoms including headache, seizures, altered consciousness, visual disturbances, and focal neurological deficits [7, 8]. While PRES is generally reversible, early diagnosis is key to reduce possible damage. Established diagnostic criteria have been lacking so far and clinical, as well as imaging findings, are often not specific. Therefore, the diagnosis of PRES can often only be made after excluding important other diagnoses. The presence of neurological symptoms of acute onset, concurrent blood pressure fluctuations, vasogenic edema as the leading neuroimaging finding, and a clinical context of associated comorbidities or trigger factors are suggestive of PRES [3]. MRI is the most important diagnostic tool and commonly shows vasogenic edema frequently following a parieto-occipital pattern [9]. Lesions in other areas such as the cerebellum, brain stem, basal ganglia, or spinal cord are less common [7].

The treatment of PRES is symptomatic since no specific therapeutic strategy is currently available. The management of the underlying disease or pathology leading to PRES development is of major importance, with reduction of blood pressure levels by 25% from baseline values to avoid acute blood pressure fluctuations [10] or elimination of the triggering factor [3]. The prognosis of PRES is mainly determined by the underlying condition since the neurological manifestations are reversible in majority of patients. However, since PRES is often accompanied by severe complications, neurological sequelae may persist, most commonly persistent epilepsy being reported [3].

Fioricet is a combination of butalbital, acetaminophen, and caffeine. It is commonly prescribed for tension headaches. When prescribed for headaches, the mechanism of action is the combination of the three compounds, acetaminophen which is analgesic, butalbital which reduces anxiety, and caffeine which augments the effects of the analgesic. The maximum dose of butalbital-acetaminophen-caffeine is 8 tablets over 24 hours, which means an equivalent of 320 mg of caffeine over 24 hours. 8 oz of a brewed cup of coffee contains 95 mg of caffeine. Espressos contain 63 mg, and decaf coffee contains 3 mg of caffeine (on average). Because of the caffeine content in butalbital-acetaminophen-caffeine, this medication has potential for causing side effects in the central nervous system and stimulating cardiovascular system, and it is recommended to be used with caution in patients with symptomatic cardiac arrhythmias [11].

## 2. Case Report

A 54-year-old right-handed Caucasian female presented to the emergency department (ED) complaining of the “worst headache of her life.” The patient reports that she took 2 tablets of butalbital-acetaminophen-caffeine every 6 hours for three days before her current presentation. Upon her presentation, her blood pressure was elevated to 178/87 mm Hg, heart rate was 76 beats per minute, respirations 18 per minute, and temperature 98.2° Fahrenheit. The patient described her headache as unilateral, involving the left periorbital area and the left occipital temporal scalp. The headache was sharp and pounding. The patient also endorsed associated sensorial changes to the left arm and gait instability. She denied any vision changes, photophobia, phonophobia, nausea or vomiting, neck pain or rigidity, or exacerbation of her headache with position changes.

The patient was seen the day before for headaches at another hospital system where she underwent head CT and lumbar puncture that were nondiagnostic. Her headache was sharp and pounding and not relieved with taking butalbital-acetaminophen-caffeine 2 tablets and tramadol 50 mg. Her blood pressure was 145/89 mmHg. During the emergency department (ED) evaluation, she was given analgesics (acetaminophen 650 mg, hydromorphone 1 mg, diphenhydramine 25 mg, and ketorolac 30 mg) that temporarily relieved her pain and was ultimately discharged home with a diagnosis of complex migraine. She was instructed to continue butalbital-acetaminophen-caffeine 50-325-40 mg, one to two tablets every six hours as needed, metoclopramide 10 mg every 8 hours, and motrin 600 mg every 6 hours.

The patient had no previous history of headaches. She had a history of hypertension, for which she was taking losartan/hydrochlorothiazide 100/12.5 mg daily, hyperlipidemia for which she was taking simvastatin 40 mg and fenofibrate 130 mg daily, anxiety on duloxetine 60 mg, and right lower extremity nerve pain for which she was taking gabapentin 100 mg every 8 hours. The patient had no history of seizures, stroke, and atrial fibrillation and denied any coffee or alcohol consumption, smoking, or other drug use.

Her initial physical exam revealed a patient in acute distress, secondary to the severe headache, but oriented to person, place, and time. She had monocular left visual field deficit. Pupils were equal, round, and reactive to light and accommodation, and extraocular movements were intact. She had abnormal cerebellar function to both finger/nose test and heel/shin test. She exhibited an abnormal, unstable gait, needing support. It was also noted that she had sensorial changes in the upper left extremity, and there was cognitive impairment to memory and concentration. The patient also had witnessed focal seizure-like activity in the ED involving left upper extremity without loss of consciousness.

**Figure 1:**
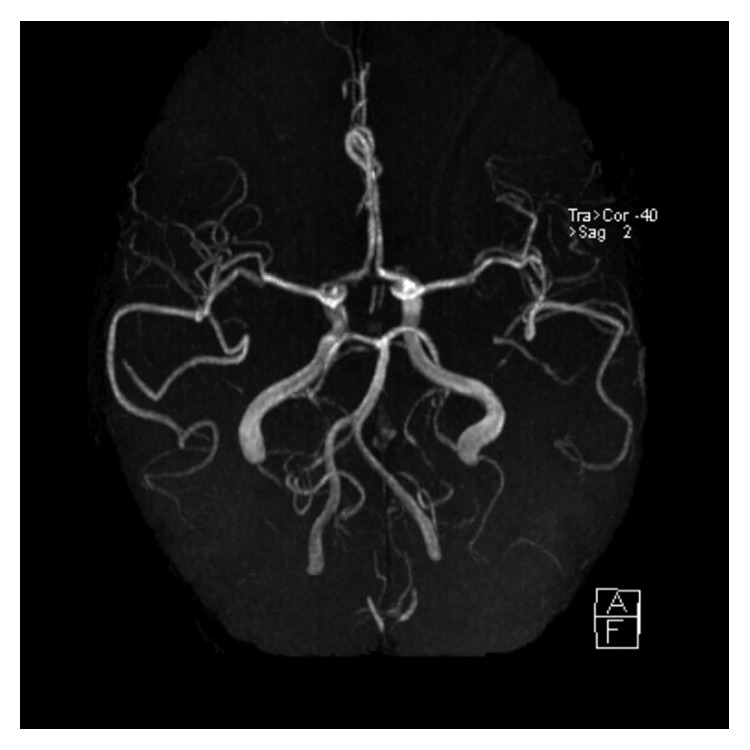
3D image: brain MRA without contrast utilizing time-of-flight (TOF) technique showing normal vasculature.

**Figure 2:**
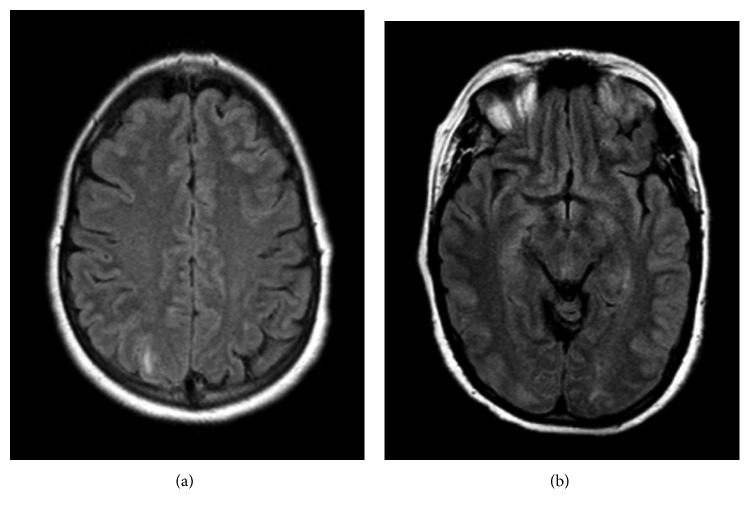
Axial FLAIR images of the brain demonstrating hyperintense signals in the (a) posterior right parietal lobe and (b) posterior occipital lobes bilaterally.

In the ED, because of persistent severe headaches, a head MRI/MRA of brain was ordered. Brain MRA was negative (Figure 1), but the MRI showed edema in the subcortical white matter of the right occipital lobe, right parietal lobe, and left occipital lobe, suggestive of posterior reversible encephalopathy syndrome (Figures2(a) and 2(b)).

**Figure 3:**
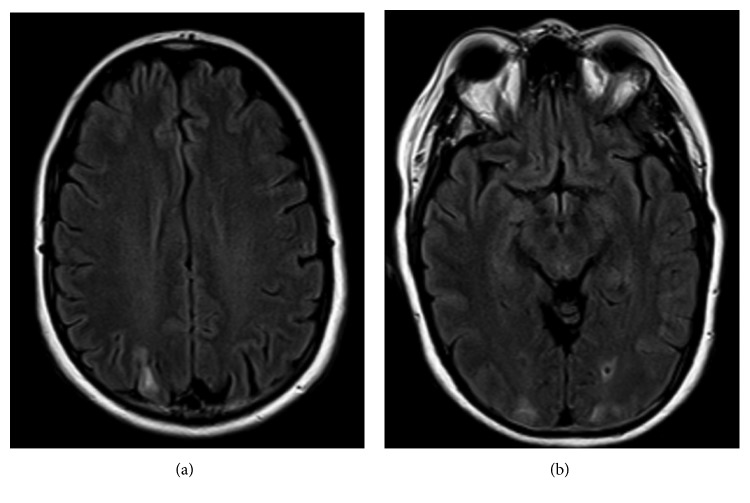
Axial FLAIR images of the brain demonstrating slight improvement of hyperintense signals in the (a) posterior right parietal lobe and (b) posterior occipital lobes bilaterally.

Twenty-four hours after being diagnosed with PRES, the patient was discharged home on topiramate 50 mg twice daily along with losartan/hydrochlorothiazide 100/25 mg daily and amlodipine 5 mg daily. The next day, she went to work and developed a severe headache. She described the pain as stabbing and hammering on her bilateral frontal areas and on top of her head and the left temporal area. The pain was worsening with any movement or exertion and it was associated with floaters and visual hallucinations of “puzzle pieces,” “squiggly lines,” and “phantom images.” She also experienced tingling in bilateral fingers. She returned to the emergency room where her blood pressure was elevated at 150/80 mmHg. Despite good control of her blood pressure during the hospital stay, the patient had persistent headaches, and five days later, her follow-up brain MRI showed that the edema in the subcortical regions of the bilateral occipital lobe has improved but not completely resolved. The edema in the subcortical right parietal lobe appeared similar to the previous study (Figures 3(a) and 3(b)). The patient was discharged on amlodipine 5 mg daily, and her losartan/hydrochlorothiazide was increased to 100/25 mg.

**Figure 4:**
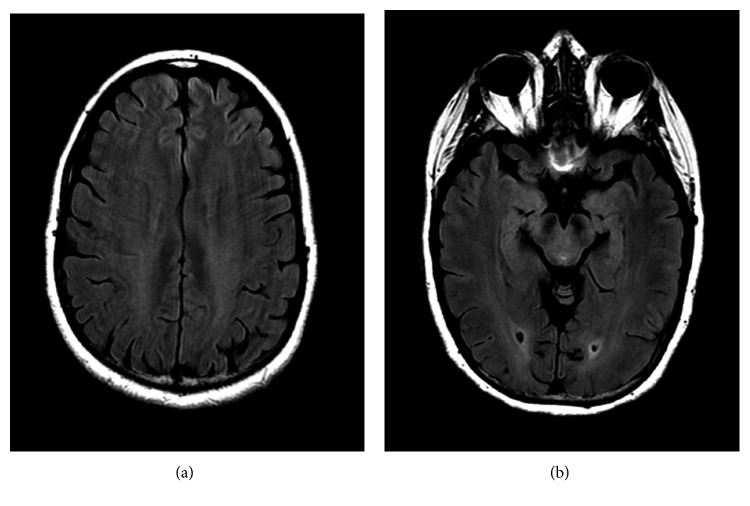
Axial FLAIR images of the brain demonstrating complete resolution of previously noted hyperintense signals in the (a) posterior right parietal lobe and (b) posterior occipital lobes bilaterally.

The patient's headaches slowly became less frequent over the next couple of months after the initial diagnosis. Her topiramate was increased to 100 mg twice a day, and the amlodipine was increased to 10 mg daily. She used hydrocodone-acetaminophen as needed for headaches. Her blood pressure was eventually controlled on atenolol 50 mg daily. Because of persistent headaches that interfered with her daily activities, her topiramate was increased to 150 mg, 2 times a day, 8 months after her initial diagnoses. On this dose, her headaches were better controlled but still recurrent. 10 months after her initial diagnosis, because of persistent headaches, despite taking topiramate 150 mg twice a day, the patient had repeat lumbar puncture that had opening and closing pressures of 15 cm/H_2_O and protein still elevated at 65 (normal 11–55). A repeat MRI showed interval resolution of previously described areas of signal abnormality that were affecting the occipital lobes and no evidence of an abnormal parenchymal signal (Figures 4(a) and 4(b)).

The patient developed clinical depression and was referred for psychiatric evaluation. She eventually was started on modafinil 100 mg daily and sertraline 50 mg daily, along with the duloxetine 60 mg and topiramate 150 mg twice a day. This combination helped control her depression for a while. Two years later, her headaches are still present, but mild at baseline. She is still using hydrocodone-acetaminophen 7.5/325 mg as needed. The patient felt that she would be unable to do her work. She stated that at times she was unable to complete tasks as before. Therefore, the patient was unable to return to work due to severe intermittent headaches. Although she was relatively quickly diagnosed and treated, the patient was unable to make a full recovery. Due to her inability to work and the constant pain, the patient still struggles with depression.

## 3. Discussion

We report a case of posterior reversible encephalopathy syndrome caused by butalbital-acetaminophen-caffeine side effects. We are attributing the side effects to the caffeine content in butalbital-acetaminophen-caffeine that resulted in subsequent elevation of blood pressure. To our knowledge, it has only been reported once before in the literature that intravenous caffeine for postlumbar puncture headaches caused PRES [12]. This patient developed immediate postinfusion blindness and generalized tonic-clonic seizures but recovered clinically and radiologically within 72 hours. Even though the onset and the symptomatology in this case was more acute and much more dramatic than in our case, the recovery was complete and very fast compared with our patient that still suffers from headaches and impaired quality of life. Interesting enough, Zappella et al. reported a duloxetine-related PRES case, where coma and myoclonus appeared a few days after starting the duloxetine treatment. The patient achieved full recovery after aggressive antihypertensive therapy and intravenous anticonvulsant therapy. Our patient has been taking duloxetine for over a year at the time of PRES diagnosis. Caffeine and duloxetine are broken down by the same enzymes in the liver: CYP450 1A2. High doses of caffeine may keep duloxetine from being removed from the body, allowing high levels to build up. Theoretically, high plasma levels of duloxetine may increase the risk of serious adverse effects such as hypertension, hypertensive crisis, increased heart rate, orthostatic hypotension, syncope, and serotonin syndrome. The combination of caffeine and duloxetine in our patient could have been the reason for the acute elevation in the blood pressure and development of PRES. The patient has been continued by her psychiatrist on duloxetine for anxiety with good clinical control and did not experience further episodes of acute elevation in blood pressure. The patient was instructed to avoid caffeine, caffeinated beverages, or butalbital-acetaminophen-caffeine.

Currently, there are no specific diagnostic criteria for PRES. The diagnosis of PRES is typically a clinicoradiological one, with more than 90% of patients having typical radiological and clinical presentations [7, 13, 14]. Most common clinical symptoms are seizures (60–75%) and less commonly patients present with altered mental status (20–25%) or headache, like in our patient (20–25%). Common diagnostic features on a MRI include white matter edema on posterior cerebral hemispheres, sparing the calcarine and paramedian parts of the occipital lobe, as well as sparing the cortical gray matter [15]. Similar to other recorded cases, our patient was mainly diagnosed using neuroimaging. The brain MRI showed edema in the right occipital, right parietal, and left occipital lobes. These results are in line with common findings associated with PRES, when over 95% of cases involve the parieto-occipital region [14]. Subsequent MRI showed no edema, which is in consensus with 81% of cases as reported by the Springer Journal of Neurology [3].

The differential diagnosis of a 54-year-old female coming with the worst headache of her life associated with left arm sensorial changes and gait instability would most commonly include acute stroke, acute aneurysmal subarachnoid hemorrhage, intracranial hemorrhage, and venous sinus thrombosis and less likely spontaneous intracranial hypotension, pituitary apoplexy, or acute myocardial infarction. Our patient's brain MRA was normal and initial EKG showed normal sinus rhythm with nonspecific *T* wave abnormalities, and serial troponins were below 0.03. Negative brain CT scan and negative lumbar puncture with the pathognomonic findings on the head MRI were eventually diagnostic for PRES.

The prognosis of PRES is generally a favourable prognosis, mainly determined by the underlying condition since most neurological symptoms are reversible in the majority of patients [16]. Appropriate reduction of blood pressure may prevent lesion progression from vasogenic edema into cytotoxic edema, infarction, or even permanent neurological findings. Unlike most cases where clinical recovery was evident before radiologic improvements were seen, our patient continued to experience symptoms (headaches) despite normalization of the brain MRI. With treatment provided in a timely manner, most patients without known preexisting conditions were able to make a full recovery; however, our patient continues to struggle with mild headaches that prevent her from making a full recovery. Known preexisting predictors of poor outcome have been identified as diabetes mellitus and corpus callosum involvement of PRES-associated lesions [17]. Recurrence is infrequent, even though trigger factors for PRES were repeatedly experienced by the patients [18]. Our patient was instructed to completely avoid any butalbital-acetaminophen-caffeine or other caffeinated compound usage in the future.

In conclusion, we report a case of PRES caused by hypertensive emergency secondary to butalbital-acetaminophen-caffeine use. We would suggest including a warning in the butalbital-acetaminophen-caffeine package regarding concerns related to adverse effects in patients with underlying hypertension.

1. Healthcare providers should be aware that butalbital-acetaminophen-caffeine can have potential severe side effects, like PRES, especially in patients with previous history of hypertension.
2. If the patients present with severe headache and associated nonfocal significant symptomatology, the threshold for further imaging should be kept very low and further diagnostic evaluation should be pursued.
3. Extremely high caffeine intake may have severe consequences; therefore, alternatives should be considered as first line in the management of headaches in patients with uncontrolled or high blood pressure.
